# Ten-year surveillance of nosocomial bloodstream infections: trends of aetiology and antimicrobial resistance in a comprehensive cancer centre

**DOI:** 10.3332/ecancer.2011.191

**Published:** 2011-02-17

**Authors:** R Passerini, TL Ghezzi, MT Sandri, D Radice, R Biffi

**Affiliations:** 1 Laboratory Medicine Unit, European Institute of Oncology, Milan, Italy; 2 Abdomino-Pelvic Surgery Division, European Institute of Oncology, Milan, Italy; 3 Department of Epidemiology and Biostatistics, European Institute of Oncology, Milan, Italy

## Abstract

**Background:**

Bloodstream infections (BSIs) are one of the major life-threatening infectious conditions in cancer patients and are responsible for prolonged hospital stays, high healthcare costs and significant mortality. Several clinical trials have reported an improved survival in patients treated with appropriate empirical broad-spectrum antibiotic therapy. Early detection of pathogens and determination of their susceptibility are essential for the optimization of treatment. Variability between hospitals is substantial and requires the individual analysis of local trends. The aim of this study is to assess the local epidemiology of BSI in a single cancer centre over a 10-year period.

**Methods:**

Retrospective microbiological surveillance of all febrile/infective episodes occurring in oncological and surgical patients in a high-volume cancer centre between January 1999 and December 2008 were considered. Patients’ data were collected, processed and analyzed using the epidemiological resource of the Virtuoso Plus software (Metafora Informatica Srl, Milano, Italy). Spearman’s rank correlation coefficient, including the two-tailed test of significance, was used to investigate trends of incidence and rate of antibiotic resistance over the 10-year period.

**Results:**

A total of 13,058 blood cultures (BCs) were performed in 2,976 patients. BCs were positive in 2,447 tests, representing 740 infective/febrile episodes: 358 (48%) in medical oncology and 382 (52%) in surgical wards. Gram-positives were responsible for the majority of episodes in oncological and surgical divisions (about 63% and 55%, respectively). Gram-positives were also the most common organism in non-catheter-related BSIs (CRBSIs) both in medical oncology (75%) and in surgical divisions (50%). *Enterococci* showed an increased resistance to levofloxacin, from 5.6% to 25.7% (*p* = 0.02) and to erythromycin, from 41.7% to 61.4%, (*p* = 0.05). Similarly, coagulase negative staphylococci (*CoNS*) developed resistance to levofloxacin and ciprofloxacin, passing from 33.9% to 67.4% (*p* = 0.01) and from 5.6% to 25.7% (*p* = 0.01), respectively.

**Conclusions:**

Gram-positives are the main pathogens of BSIs; there is no difference in aetiology of CRBSIs between surgical and oncological patients. The lower incidence of gram-positive non-CRBSIs in surgical patients was probably due to gram-negative infections secondary to surgical complications.

## Background

Bloodstream infections (BSIs) are one of the major life-threatening infectious conditions in cancer patients, responsible for prolonged hospital stays, high healthcare costs, and significant mortality [1–4]. The worldwide rising incidence of nosocomial BSI is mainly attributed to the increased use of invasive devices, increased frequency of invasive procedures and increased use of aggressive drug therapy resulting in immunodeficiency [1–3]. In the past, the BSI had a common global trend: infections from gram-negative organisms predominating during the 1970s, while gram-positives became the most common pathogens in the 1980s [1]. More recently some authors have suggested a possible reversal of this trend [5–7]. Moreover, several studies have indicated that many organisms that cause hospital-acquired BSI are becoming resistant to antibiotics [1–3,6,8,9]. Whereas several clinical trials have reported an improved survival of patients treated with appropriate empirical broad-spectrum antibiotic therapy [2,5,6,9–11]. Early detection of pathogens and determination of their susceptibility are essential for the optimization of treatment [12]. Variability between hospitals is substantial, and requires the individual analysis of local trends. Therefore, nosocomial surveillance is essential to decrease morbidity and mortality of nosocomial BSI within these structures [2,5,6,9–11]. The aim of this study is to assess the local epidemiology of BSI in a single cancer centre over a 10-year period.

## Methods

### Setting, patients and study design

The current retrospective study included all consecutive blood cultures (BCs) performed in the European Institute of Oncology, Milan, Italy, between January 1999 and December 2008. Patients and laboratory information were retrieved from a computerized hospital database. All the patients signed an informed consent form which allowed the use of their data in future scientific studies. The sample studied consisted of all patients admitted to either the surgical divisions or the medical oncology division. Those admitted to the surgical divisions were mainly immunocompetent subjects undergoing treatment for primary and metastatic tumours. Patients admitted to the division of medical oncology were predominantly immunosuppressed subjects undergoing chemotherapy for various solid tumours, while those from the division of haematology–oncology were receiving aggressive chemotherapy or high-dose chemotherapy with autologous peripheral blood stem cell transplantation for leukaemia, lymphoma or solid tumours. Some patients required a short-term or a long-term central venous catheter (CVC) for antibiotic or chemotherapy administration or parenteral nutrition during their hospital stay.

### Microbiology studies and definitions

For each patient only one BC per organism per febrile/infective episode was included in our study. A second episode occurring after 30 or more days or during another hospital stay was considered as a separate case. For the diagnosis of catheter-related BSIs (CRBSIs), three BCs were collected at the onset of fever: two from the CVC—taken one hour apart from the other—and one from a peripheral vein. Febrile/infective episode was defined as a single body temperature peak above 38.5 °C or a sustained body temperature of more than 38.0 °C for two observations within 24 hours and not being a consequence of the administration of potentially pyrogenic agents. For the diagnosis and/or the confirmation of non-CRBSIs, the BCs were performed on two peripheral venipuncture samples collected in duplicate with a one hour interval [13,14]. Isolation of coagulase negative staphylococci (*CoNS*) was considered significant only if detected from multiple cultures or from a clinically compatible event. We considered the resistance to cephalosporins as a possible marker for extended-spectrum β-lactamase (ESBL) [15]; data relating to ESBL were only available in cases diagnosed after 2006.

### Laboratory analysis

A 20 ml blood sample was obtained aseptically and divided equally between BACTEC aerobic/F resin and a BACTEC lytic/10 anaerobic/F bottles (Becton Dickinson, Sparks, MD, USA). The bottles were incubated in the BACTEC 9050 Instrument (Becton Dickinson, Sparks, MD, USA) at 35 °C, using a 5-day protocol for bacteria (according to the manufacturer’s recommendations) and a 10-day protocol for fungi. In all the positive cultures, time to positivity (TP), defined as the time from incubation to a positive result, was recorded. An aliquot of the blood-broth mixture from those patients with positive cultures was used for a preliminary Gram stain identification and the remaining amount for the direct antibiogram following the Kirby–Bauer method (according to the result of the Gram stain). Identification and antimicrobial susceptibility testing were performed using the Microscan Walk-Away (Dade Behring SpA) for aerobic gram-positive and gram-negative organisms, the Cristal (Becton Dickinson, Sparks, MD, USA) for anaerobic bacteria and the API AUX (bioMerieux) for fungi. The diagnosis of CRBSIs was based on the differential time to positivity [16–18]. Clinical assessments were performed from time to time by the microbiologist and the medical staff to determine the clinical relevance of organism which is isolated. Patients’ data were collected, processed and analyzed using the epidemiological resource of the Virtuoso Plus software (Metafora Informatica Srl, Milano, Italy).

### Statistical analysis

To investigate trends of incidence and rate of antibiotic resistance over the years, the Spearman’s rank correlation coefficient (r_s_) was used and its significance was calculated using a two-tailed t-approximation the statistical software MedCalc release 8.2.0.3 (MedCalc software, Mariakerke, Belgium) and Stata/SE 11.0 (StataCorp, College Station, TX, USA) were used for the statistical analysis of all data. *P*-values of 0.05 or less were considered to be statistically significant.

## Results

During the 10-year microbiological surveillance, a total of 13,058 BCs were performed for 4,308 febrile episodes occurring in 2,976 patients; 2,861 (66.4%) episodes were possible CRBSIs and 1,447 (33.6%) were possible non-CRBSIs. The mean overall number of BC sets collected for each suspicious BSI was 3.03 (13,058 BCs for 4,308 episodes) as recommended by internal guidelines. BCs were positive in 2,447 tests, representing 740 febrile/infective episodes. Patients of the medical oncology divisions and the surgical divisions experienced 358 (48%) and 382 (52%) episodes, respectively. Among surgical divisions, the BSIs were most commonly reported in general/abdomino-pelvic (42%), gynaecological (18%) and thoracic surgery (11%).

**Figure 1:**
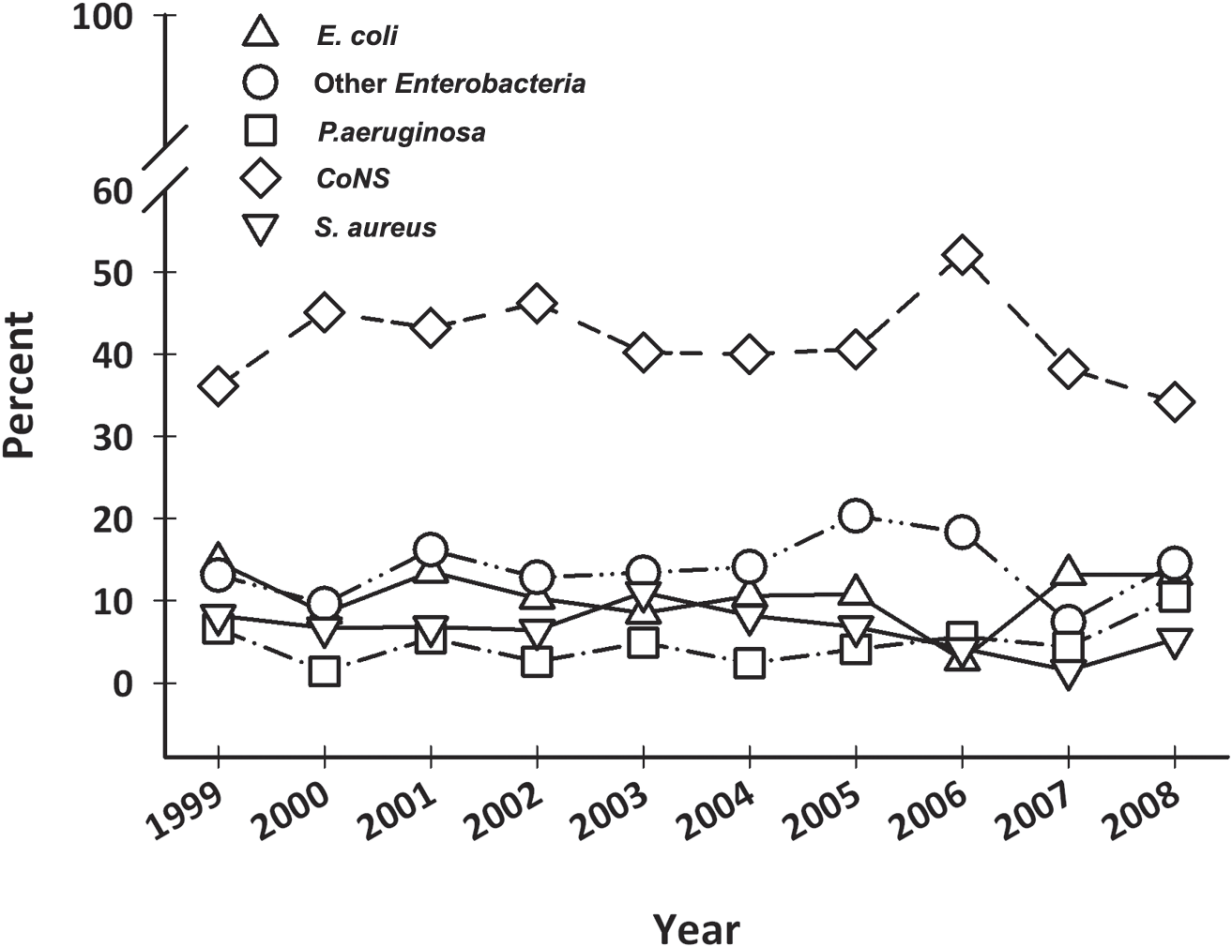
Main isolates in blood culture from 1999 to 2008 (%). CoNS: coagulase negative staphylococci.

Data concerning BCs performed in the surgical divisions and in the medical oncology divisions are summarized in Table 1. Gram-positive organisms were responsible for the majority of episodes both in oncological and surgical wards (about 63% and 55%, respectively); anaerobes represent about 3% of the BSIs; while fungi and other organisms (unusual microorganisms or polymicrobial growth) represent about 2%. Gram-positives were the most common organisms among CRBSIs both in oncological and surgical subjects (about 62% and 58%, respectively). Gram-positives were also the most common organisms in non-CRBSIs both in medical oncology divisions (75%) and surgical divisions (50%). More than 40% of the pathogens were *CoNS* and about 20% were *Enterobacteria*—mainly *Escherichia coli* (10%) and *Klebsiella* species (6%). *Staphylococcus aureus* was responsible for 7% and *Pseudomonas aeruginosa* for 5% of BSIs. Figure 1 and Table 2C show the main organisms isolated over the years. Only *S. aureus* showed a statistically significant reduction in incidence rate (*p* = 0.04), whereas the incidence of the other agents did not change.

**Figure 2:**
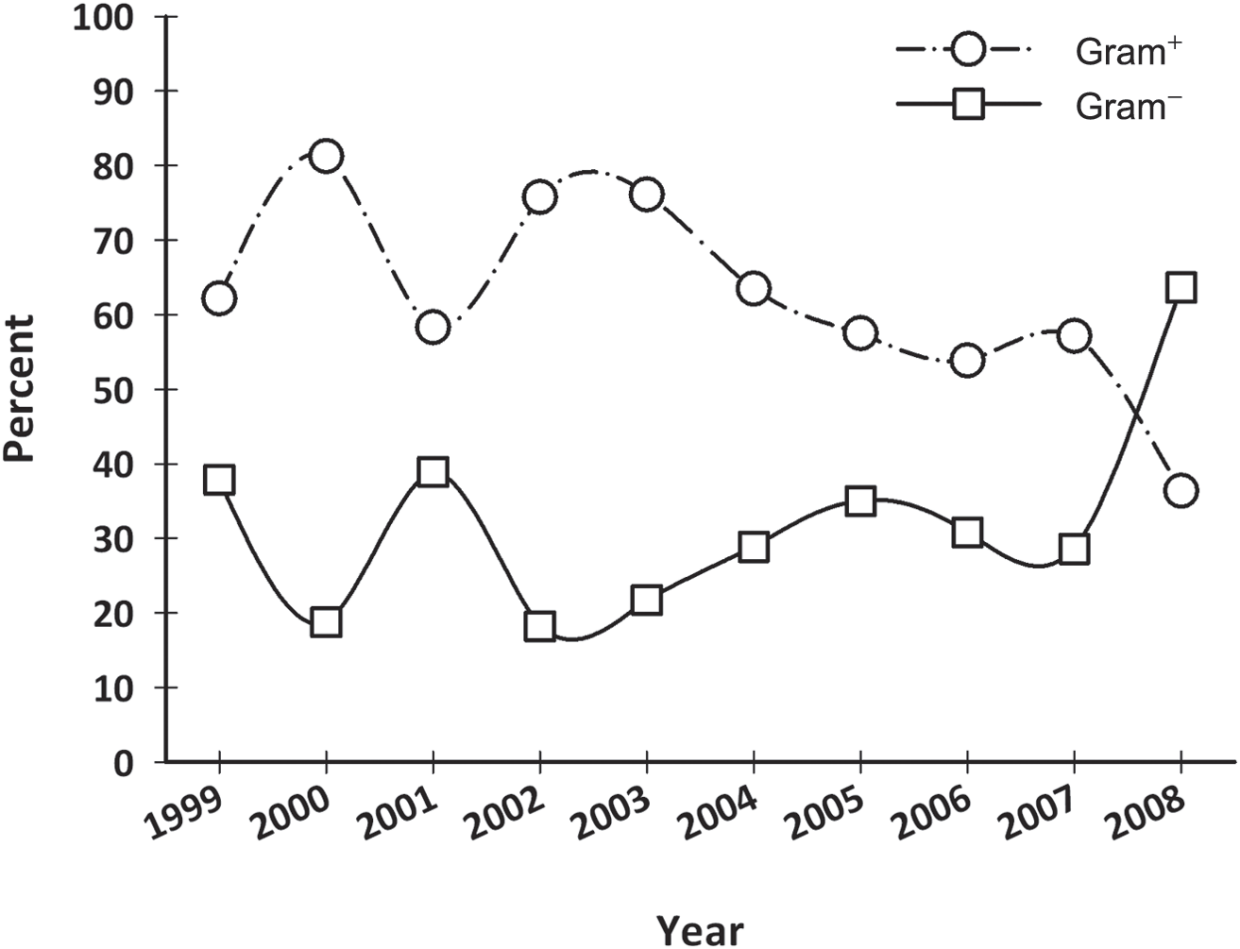
Gram-positives and gram-negatives isolated in blood cultures from medical oncology patients from 1999 to 2008 (%).

Considering the percentage of BSIs arising from gram-positive and gram-negative bacteria over the years, a statistically significant decrease in the incidence of gram-positive infections was observed in oncological patients in 2008, from 57% to 36% (*p* = 0.03) (Figure 2 and Table 2C). Despite this fact, there was no statistically significant difference over the years, in all patients or in surgical patients.

The overall rate of BCs performed was 78 per 1,000 hospital admissions while the BSI incidence was 4.4 per 1,000. About 3.5 were CRBSIs and 0.9 were non-CRBSIs. A statistically significant decrease in the incidence of BCs performed (from 104.0 to 71.8, r_s_ = −0.87, *p* = 0.001), suspicious infective episodes (from 34.7 to 25.0, r_s_ = −0.86, *p* = 0.001) and CVC-related infections (from 25.3 to 14.6, r_s_ = −0.58, *p* < 0.001) was observed in overall patients. Table 2A shows the annual incidence (per 1,000 hospital admissions) of BCs and isolates from 1999 to 2008. A statistically significant decreasing trend in the incidence of BCs performed (from 210.7 to 72.2, r_s_ s = −0.89, *p* < 0.,001) was also observed among oncological patients. The incidence of suspicious infective episodes (from 36.2 to 12.2; r_s_ = −0.82, *p* = 0.004), BSIs (from 8.6 to 3.9; r_s_ = −0.72, *p* = 0.019) and CRBSIs (from 50.3 to 15.3; *p* = 0.002) also decreased among these patients (Table 2B).

A statistically significant increase in the incidence of BCs performed was observed among surgical patients (from 29.9 to 71.4; *p* = 0.02). No other statistically significant difference was observed in this group.

With regard to antimicrobial resistance, over the last 10 years, *Enterococci* have showed an increased resistance to levofloxacin and to erythromycin from 5.6% to 25.7% (r_s_ = 0.86, *p* = 0.02) and from 41.7% to 61.4% (r_s_ = 0.64, *p* = 0.05) respectively; *CoNS* have developed an increased resistance to levofloxacin (from 33.9% to 67.4%, r_s_ = 0.97, *p* = 0.01) and to ciprofloxacin (from 5.6% to 25.7%, r_s_ = 0.84, *p* = 0.01); *Enterobacteriaceae* developed an increased resistance to gentamicin (*p* = 0,05), although without alarming values (from 1.2% to 9.7%). Other trends in resistance were not statistically significant e.g. both ESBL organisms and *Pseudomonas* species did not show a statistically significant difference.

## Discussion

This study was designed to assess BSIs in a single cancer centre, to gain data on epidemiology and *in vitro* antibiotic susceptibility which can be used to improve our internal guidelines for BSIs and to allow comparison with other centres. First, we should underline the fact that the methods of microbiological analysis used in our laboratory did not change during the period of this study. In spite of this, from April 2002 the Differential Time to Positivity for diagnosis of CVC-related bacteraemia [16–18] was introduced in routine analysis, with a consequent decrease in the number of CVC cultures performed. Despite an expected increase in the number of BCs performed for the diagnosis of CVC-related infections, this increase was only observed in surgical wards as a consequence of increased adherence to internal guidelines (which recommends at least 3 samples for each suspicious infective episode).

In agreement with the literature [1,19], nearly two thirds of episodes were caused by gram-positive organisms, both in surgical and oncological patients. The most frequent organisms were *CoNS*, *E. coli*, *P. aeruginosa*, other *Enterobacteria* and *S. aureus*, also consistent with other reports [15,20,21]. The distribution of gram-positive and gram-negative CRBSIs was similar. On the other hand, in subjects from the medical oncology wards, gram-positive bacteria were the most common cause of non-CRBSIs. In 2008 a peak incidence of gram-negative BSI of 63.4% (2.49/1,000 vs. 1.42 /1,000 hospital admissions) in the medical oncology wards, motivating an alteration to the antibiotic prophylaxis protocols [22]. Although uncommon, anaerobes and fungi were more frequent in surgical subjects, unusual microorganisms and polimicrobial growth have been reported in oncological patients.

Regarding the antimicrobial susceptibility profile, an important finding was the statistically significant increased resistance of gram-positives, mainly *CoNS*, to quinolones. *CoNS* resistance to oxacillin was constantly high for all the surveillance period, while the incidence of *S. aureus* was unstable. An increased resistance to gentamicin was reported among *Enterobacteria,* in particular *E. coli* developed an increased resistance to ampicillin (from 47.4% to 68.9%). Although an increase in the number of BSIs due to ESBL organisms has been reported by other authors, ESBL was not a concern in our surveillance [15,23–25].

Nonfermentative gram-negatives had a good susceptibility to antibiotics while *P. aeruginosa* showed a statistically significant decrease in resistance. During the 10-year study period only nine strains of *Acinetobacter* were isolated (one *Acinetobacter baumannii* and eight *Acinetobacter lwoffii*). Although it demonstrated high resistance to cephalosporin (both 1st and 2nd generation) there was no statistically significant difference over the years.

Our study has some limitations. First, despite the extensive review of BCs performed and the long period of surveillance examined, conclusions are based on a retrospective analysis of laboratory data. Second, there is a lack of data evaluating the ESBL production during the period from 1999 to 2005, when this information was not available.

## Conclusion

Gram-positives were the main pathogens in nosocomial BSIs, both in surgical and oncological patients. There was no difference in aetiology of CRBSIs between surgical and oncological patients. The lower incidence of gram-positive non-CRBSIs among surgical subjects was probably due to prevalent gram-negative infections, secondary to post-operative complications of abdomino-pelvic, gastrointestinal, gynaecological and urological surgeries [26]. Antibiotic resistance was not a serious concern owing to a careful policy of nosocomial surveillance, adopted institutional guidelines for antibiotic prophylaxis and to early empiric treatment of infectious complications [27].

## Figures and Tables

**Table 1:** Blood cultures data

	**Overall (%)**	**Medical oncology patients (%)**	**Surgical patients (%)**
All BCs performed	13,058	7,050	6,008
Suspicious infective episodes	4,308 (100)	1,929 (100)	2,379 (100)
Number BC/infective episodes	3.03	3.65	2.52
Patients	2,976	1,047	1,929
CVC-related infections	2,861 (66.4)	1,598 (82.8)	1,263 (53.1)
Non CVC-related infections	1,447 (33.6)	331 (17.2)	1,116 (46.9)
Negative BC	3,568 (82.8)	1,571 (81.4)	1,997 (83.9)
Positive BC	740 (17.2)	358 (18.6)	382 (16.1)
Gram-positive	436 (58.9)	225 (62.8)	211 (55.2)
Gram-negative	255 (34.5)	116 (32.4)	139 (36.4)
Anaerobes	20 (2.7)	7 (2)	13 (3.4)
Fungi	15 (2)	1 (0.3)	14 (3.7)
Other pathogens	14 (1.9)	9 (2.5)	5 (1.3)

BC, blood cultures; CVC,central venous catheter.

**Table 2A:** Annual incidence (per 1000 admissions) of BCs and isolates, 1999–2008, whole series

	**1999**	**2000**	**2001**	**2002**	**2003**	**2004**	**2005**	**2006**	**2007**	**2008**	**r_s_**	***P* for trend**
All BCs performed	104.0	104.6	86.2	83.1	75.4	74.6	62.4	67.9	70.0	71.8	−0.87	*0.001*
Suspicious infective episodes	34.7	35.8	29.7	26.7	29.6	25.5	22.4	21.2	25.5	25.0	−0.86	*0.001*
BC/infective episodes	3.2	3.0	3.1	3.4	2.6	3.0	2.9	3.4	2.8	3.0	−0.31	0.377
Patients	22.9	23.9	19.6	18.3	22.6	18.7	15.9	16.7	19.9	18.6	−0.58	0.082
CVC-related infections	25.3	26.5	20.5	16.8	17.4	16.6	14.5	12.4	13.0	14.6	−0.89	*<0.001*
Non CVC-related infections	9.5	9.3	9.2	9.9	12.2	8.9	7.9	8.7	12.4	10.4	0.11	0.751
Negative BC	29.0	30.0	24.6	21.9	24.2	20.3	18.4	17.6	21.7	20.8	−0.79	*0.006*
Positive BC	5.8	5.5	5.1	4.8	5.4	5.2	4.0	3.6	3.6	4.2	−0.83	*0.003*
Gram-positive	3.2	3.9	3.1	3.0	3.6	3.0	2.3	2.1	1.9	2.1	−0.87	*0.001*
Gram-negative	2.6	1.4	1.8	1.5	1.6	1.5	1.5	1.9	1.0	1.9	−0.09	0.790
Anaerobes	0.0	0.0	0.1	0.2	0.2	0.4	0.0	0.1	0.2	0.1	0.33	0.346
Fungi	0.1	0.1	0.1	0.1	0.1	0.1	0.0	0.1	0.2	0.2	0.45	0.192
Other pathogens	0.0	0.1	0.0	0.0	0.1	0.1	0.2	0.2	0.3	0.0	0.48	0.157

BC, blood culture; CVC, central venous catheter.

*p*-values <0.05 are italicised.

**Table 2B:** Annual incidence (per 1,000 admissions) of BCs and isolates, 1999–2008, medical oncology patients

	**Year**										**r_s_**	***P* for trend**
	**1999**	**2000**	**2001**	**2002**	**2003**	**2004**	**2005**	**2006**	**2007**	**2008**		
All BCs performed	210.7	159.6	120.6	92.7	88.9	80.4	63.7	48.1	65.1	72.2	−0.89	*< 0.001*
Suspicious infective episodes	36.2	26.6	17.9	14.5	15.6	25.4	10.7	10.1	12.6	12.2	−0.82	*0.004*
Patients	61.8	46.0	31.7	23.9	27.2	25.4	19.9	14.3	19.5	20.3	−0.88	*< 0.001*
CVC-related infections	50.3	38.6	25.2	18.8	20.5	19.4	14.8	10.9	14.9	15.3	−0.85	*0.002*
Non CVC-related infections	11.5	7.4	6.5	5.9	6.7	6.0	5.0	3.3	4.5	5.0	−0.87	*< 0.001*
Negative BC	53.5	40.8	26.6	19.6	20.7	18.9	15.3	11.3	16.3	16.4	−0.88	*< 0.001*
Positive BC	8.6	5.3	5.0	4.3	6.4	6.5	4.6	3.1	3.2	3.9	−0.72	*0.019*
Gram-positive	5.3	4.3	3.0	3.4	4.9	4.1	2.6	1.7	1.8	1.4	−0.84	*0.022*
Gram-negative	3.2	0.1	1.8	0.7	1.4	1.9	1.6	0.9	0.9	2.5	0.04	0.920
Anaerobes	0.0	0.0	0.1	0.2	0.0	0.2	0.0	0.1	0.1	0.0	0.10	0.786
Fungi	0.0	0.0	0.0	0.0	0.1	0.0	0.0	0.0	0.0	0.0	−0.06	0.873
Other pathogens	0.0	0.0	0.0	0.0	0.0	0.2	0.3	0.4	0.3	0.0	0.59	0.073

BC, blood culture; CVC, central venous catheter.

*p*-values <0.05 are italicised.

**Table 2C:** Percent of gram-positive, gram-negative and main isolates in BC of medical oncology patients

	**Year**										**r_s_**	***P* for trend**
	**1999**	**2000**	**2001**	**2002**	**2003**	**2004**	**2005**	**2006**	**2007**	**2008**		
Gram-positive	62.2	81.2	58.3	75.8	76.1	63.5	57.5	53.9	57.1	36.4	−0.75	*0.013*
Gram-negative	37.8	18.8	38.9	18.2	21.7	28.9	35.0	30.8	28.6	63.6	0.22	0.533
*E. coli*	14.7	8.5	13.5	10.3	8.5	10.6	10.8	2.8	13.2	13.2	−0.12	0.750
Other *Enterobacteria*	13.1	9.1	16.2	12.8	13.4	14.1	20.3	18.3	7.4	14.5	0.28	0.425
*P. aeuroginosa*	6.6	1.4	5.4	2.6	4.9	2.4	4.1	5.6	4.4	10.5	0.25	0.489
*CoNS*	36.1	45.2	43.2	46.2	40.2	40.0	40.6	52.1	38.2	34.2	−0.24	0.511
*S. auerus*	8.2	6.7	6.8	6.4	11.0	8.2	6.8	4.2	1.5	5.3	−0.57	0.083

CoNS, coagulase negative staphylococci.
